# Attentional bias to infant faces might be associated with previous care experiences and involvement in childcare in same-sex mothers

**DOI:** 10.1016/j.ijchp.2023.100419

**Published:** 2023-10-23

**Authors:** Micol Gemignani, Michele Giannotti, Paola Rigo, Simona de Falco

**Affiliations:** a Department of Psychology and Cognitive Science, University of Trento, Rovereto 38068, TN, Italy; b Department of Developmental Psychology and Socialisation, University of Padova, Padova 35131, PD, Italy

**Keywords:** Attention, Parenting, Same-sex mothers, Involvement, Parental acceptance–rejection

## Abstract

**Background:**

Attentional bias toward infant faces is associated with parental sensitivity and supports the infant-caregiver attachment relationship, ultimately fostering child health outcomes. However, experience-related determinants of parents' attentional bias to infant faces have been poorly investigated. We examined attentional bias to infant versus adult faces in a sample of same-sex mothers (*N* = 76), and whether it varied depending on maternal involvement in childcare and the perceived quality of past experiences of care.

**Method:**

A Go/no-Go attentional task was used to compare the effects of infant and adult faces in retaining attention. Maternal involvement in childcare was measured using items addressing nurturing behaviors. Memories of past experiences of care were collected using the short-form version of the Parental Acceptance-Rejection scale.

**Results:**

Results confirmed that infant faces induced greater attentional bias compared to adult faces. More involved mothers were more biased, in terms of attention, to infant versus adult faces. Attentional bias to infant versus adult faces increased as mothers felt more rejected by their own fathers during childhood.

**Discussion:**

Our findings suggested that attentional bias to infant faces might be associated with past experiences of care and direct commitment in childcare in same-sex mothers. Robust and accurate empirical findings on same-sex parent families are essential to inform social policies supporting these families’ well being.

## Introduction

Parent–infant interactions rely on the ability to express signals through facial expressions (Ainsworth & Bell, 1970; Ainsworth et al., 1978). In particular, infants’ nonverbal signals capture the attention of adults and are used to communicate needs (Lucion et al., 2017), with the evolutionary goal to get care and protection from caregivers (Lorenz, 1943). Appropriate perception and interpretation of infants’ signals is an integral part of sensitive caregiving, which supports the development of a secure infant–caregiver attachment relationship (Ainsworth et al., 1978). Given the documented implications on the quality of parent–child bonding (Dudek & Haley, 2020; Pearson et al., 2011a), a specific line of research examined maternal attention to infant cues.

### Attentional bias toward infant faces

Behavioral tasks assessing attentional bias in parents typically present infant cues as distractors during conflict tasks (e.g., Stroop, Go/No Go, visual search tasks). The attentional bias index refers to the difference in attention captured by distinct stimuli (e.g., infant versus adult faces), which suggests a greater cognitive engagement to a particular stimulus. Using an attentional Go/no-Go task, Pearson and colleagues (2010) demonstrated that pregnant women took longer to respond to the peripheral stimuli when infant faces, in particular those displaying distress, appeared on the screen as distractors. In a following study (Pearson et al., 2011a), a greater attentional bias toward infant distressed faces was associated with a more successful mother-infant bonding at 3–6 months postpartum. Using a modified Irrelevant Feature Visual Search paradigm (Theeuwes, 1991) Thompson-Booth and colleagues (2014a) demonstrated that women, mothers in particular, showed slower Reaction Times (RTs) in search arrays containing infant versus adult faces. The highly salient nature of infant faces elicited greater attention compared to pre-adolescent, adolescent, or adult faces in mothers versus non-mothers (Thompson-Booth et al., 2014b).

### The role of maternal experiences in caring for a child

Psychological characteristics of mothers have been found to modulate their attentional bias to infant faces (Lucion et al., 2017). Pearson and colleagues (2010) demonstrated that whilst non-depressed pregnant women took longer to disengage attention from distressed infant faces, this was not detected in women experiencing depressive symptoms. Thompson-Booth and colleagues (2014a) found that a greater attentional bias to infant faces was associated with less parental distress reported by mothers. In addition, a greater attentional bias to infant versus adult faces was found in high- versus low-sensitive mothers (Dudek & Haley, 2020). On the other hand, little attention has been put on how attentional bias to infant faces is related to maternal experiences in caring for their child. Overall, a higher engagement of attention toward infant distressed faces was found in breastfeeding compared to formula-feeding mothers (Pearson et al., 2011b). Pearson and colleagues (2010) demonstrated that the attentional bias towards distressed infants was greater in multiparous compared to primiparous women. Whilst the effect of parity in modulating maternal responses to infant faces has been corroborated using other methodologies (e.g., Event-Related Potentials; Maupin et al, 2019; Rutherford et al., 2019), different nuances of caregiving experiences have been mainly neglected. In addition, behavioral research concerning adoptive parents or surrogate parents, which would complement the current knowledge about experience-related determinants of the attentional bias to infant cues, is currently lacking. To date, only one research has provided partial evidence on the role of early parental involvement in childcare in the attentional bias to infant faces. In a sample of different-sex parents, Gemignani and colleagues (2022) reported that parents’ sex did not have a significant effect on the attentional bias to infant faces when considering the variability explained by the amount of parental involvement in early childcare. However, the gendered division of childcare in different-sex couples of parents might have made it difficult to distinguish between the role of sex and involvement in modulating parental responsiveness (Giannotti et al., 2022a), as mothers frequently result to be more involved in early childcare as compared to fathers (e.g., Gemignani et al., 2022).

### The role of maternal experiences of being cared for as a child

The attentional bias toward infant faces might be also linked to the perceived quality of care received during childhood. According to Bowlby's theoretical work (1969/1982), repeated interactions with attachment figures are schematized in the form of Internal Working Models (IWM) that synthesize the main self-other interactive dynamics and are automatically activated by individuals when processing new situations. Similarly, in the theoretical framework of the Interpersonal Acceptance-Rejection Theory (IPARTheory; Rohner, 2016), it has been assumed that the perceived interpersonal rejection during childhood may hinder the development of stable mental representations, which constitute a view of self, others and interpersonal contexts (Rohner, 2016). At the empirical level, retrospective remembrances of care experiences during childhood have been found associated with parents’ implicit and explicit responses to infant faces (Gemignani et al., 2022; Senese et al., 2018). It has been demonstrated that parents who reported a more accepting maternal care were more biased, in terms of attention, by infant versus adult faces (Gemignani et al., 2022). Though evidence is still scarce, it overall suggests that early care experiences with one's own mothers might shape implicit attentional responses to infant cues. Differently, very scarce evidence has been provided regarding the role of the perceived quality of early paternal care (Khaleque & Rohner, 2002).

### Same-sex parenting

Despite constituting an antecedent of maternal sensitivity and mother–child relationships (Dudek & Haley, 2020; Pearson et al., 2011a), research on attentional bias toward infant faces have been confined to heteronormative samples of mothers. Up to date, there is no specific evidence about the attentional bias to infant faces and its associated factors in same-sex parent families. Besides extending empirical knowledge on parenting beyond the heteronormative perspective, it should be noted that investigating the correlates of attentional bias in same-sex parent families could be advantageos, as it might shed further light on the underlying mechanisms of human caregiving behaviors. About the role of maternal involvement in childcare, it has been consistently evidenced that same-sex mothers share parenting more equally than do mothers and fathers within different-sex parent families (Patterson et al., 2004; Patterson, Riskind, & Tornello, 2013). This might result in individual differences in maternal involvement that are not largely defined according to the traditional sex roles as in different-sex couples of parents, in which mothers usually devote much time in childcare as compared to fathers. When it comes to the contribution of the perceived quality of care during childhood, the experience of parental rejection itself might somewhat differ among sexual minorities, and they might experience more early adverse events related to stigmatization. This topic should require greater attention, since the link between parental rejection, psychological adjustment, and personality development has proved even stronger among sexual minorities than in the general population (Ryan et al., 2009; 2010). That being said, potential variation in the attentional bias to infant faces might reflect a greater developmental exposure or sensitivity to poor environmental experiences with parental early care in same-sex parent families. Overall, it has been suggested that the empirical knowledge related to IPARTheory needs to be developed further to encompass LGBTQIA+ people experiences (Fuller, 2017). However, no research to date has investigated the role of recollected experiences of care in the attentional bias to infant faces in same-sex parent families.

### The current study

The main goal of our study is to clarify the contribution of experience-related factors on the attentional bias to infant cues in same-sex mothers. Therefore, we primarily aim to i) confirm that infant faces retain more attention compared to adult faces; ii) investigate whether the attentional bias to infant versus adult faces is associated with mothers’ involvement with childcare; iii) investigate whether individual variations in past experiences of care with mothers' own caregivers are associated with the attentional bias to infant versus adult faces. First (i), we expect that infant faces interfere with the task performance more than adult faces, slowing RTs to peripheral stimuli in Go conditions. Given that an attentional bias to infant distressed faces has been specifically found in previous research (Pearson et al., 2010; 2011a; 2011b; 2013), we might also expect to find an interaction effect between the face age (infant versus adult) and emotional valence (happy, neutral, sad). Secondly (ii), we expect that the attentional bias to infant versus adult faces could be related to maternal commitment in childcare. Thirdly (iii), we expect that the experience of receiving care from one's own mother during childhood might be related to the attentional bias to infant faces. Due to scarce previous evidence regarding the contribution of paternal care, we are cautious in putting forward a priori hypotheses regarding its role in the attentional bias to infant faces.

## Methods

### Participants

A group of *N* = 76 mothers being in a same-sex couple participated in the study. Contact with most mothers was made through the Italian Association “*Famiglie Arcobaleno*” (i.e., an association that brings together same-sex parents in Italy), which sent an invitation to participate in the study to all its members through the mailing list. A snowball sampling method was also used, such that mothers who participated in the study were asked to forward the study invitation to other same-sex mother families who might be interested in joining the study. To be included in the study sample, i) the age of mothers’ only or youngest child should range between 2 and 36 months; ii) mothers raised their child since birth. Both members of each same-sex couple were first invited to participate in the study together. However, whilst 70 mothers (92 %) participated in the experiment with their partner, 6 mothers (8 %) participated alone. Only participants who completed the whole experimental procedure (*n* = 73 mothers) were included in the final analyses; whilst two mothers did not complete the task, one mother was excluded from the final sample for technical issues while performing the task. The majority of participants were Italian (95.8 %), but 3 of them (4.2 %) reported to be of a different nationality (i.e., German, American, Ecuadorian). The Socio-Economic Status (SES) was calculated according to Hollingshead's (1975) criteria (Rossi, 1994). An overview of study participants is reported in Table 1. The study was approved by the ethical committee of the University of Trento and complied with the Helsinki declaration.

**Table 1:** Descriptive characteristics of the study participants; N = number; SD = standard deviations.

Variable	*N*	Mean or Percentage	SD
**Socio-Economic Status (SES)**	73		
**medium-low**	4	5.5 %	
**medium**	16	22 %	
**medium-high**	31	42.5 %	
**high**	22	30 %	
**Parent age**	73	39.1	5.7
**Nationality**	73		
**Italian**	70	95.8 %	
**Non-Italian**	3	4.2 %	
**Relationship with partner**	73		
**<5 years**	10	13.6 %	
**6–10 years**	36	49.4 %	
**11–15 years**	21	28.8 %	
**>15 years**	6	8.2 %	
**Number of children**	73		
**Primiparous (1 child)**	63	86.3 %	
**Multiparous (2 children)**	10	13.6 %	
**Child age (in months)**	73	17.4	11.8
**Involvement in childcare**	71	43.7	5.1
**PARQmother**	73	38.8	12.8
**PARQfather**	72	46	15.2

### Instruments

Sociodemographic data was collected. Mothers were asked to answer all the questions by referring to the youngest child in case of multiple children.

#### Parental involvement in childcare

To assess parental involvement in childcare, mothers completed 10 items (Supplementary Materials) adapted from the Parental primary caregiving role Structured Interview (Abraham et al., 2014). The original set of questions covered multiple caregiving domains, including parental responsibilities (e.g., take the child to the doctor), nurturing (e.g., change the diaper, prepare the bottle) and playful behaviors (e.g., tickle the child, blow on their belly). For this study, we considered only the questions related to parental nurturing behaviors, such as those behaviors that parents exhibit in daily contact with the child. Of note, the selected pool of items included activities which might be accomplished by both recognized and unrecognized parents in the Italian context. Items have been translated into Italian and back-translated in English. To compute a total score, responses to items (1 = *not at all;* 2 *=* *rarely;* 3 *=* *a few times a week;* 4 *=* *About once a day*; 5 = *more than once a day*) were summed, with a higher score reflecting a higher degree of involvement. Cronbach's alpha was satisfactory (*α* = 0.81). The McDonald's ω total was 0.87.

#### Recollected experiences of care

Mothers completed the Italian validated short-form version (Senese et al., 2016) of the Parental Acceptance-Rejection scale (PARQ) (Rohner, 2005b). According to the IPARTheory (Rohner, 2016), all interpersonal relationships with significant others are characterized by an affectional bond that falls somewhere along a continuum from acceptance to rejection. The Italian validated short-form version of PARQ consists of two scales measuring past experiences of care with one's own mother and father. Each scale, which has 24 items, originates a total maternal/paternal score (i.e., PARQmother and PARQfather) consisting of four different dimensions: (1) warmth/affection, (2) hostility/aggression, (3) indifference/neglect, and (4) undifferentiated rejection. Computing the total score (high score = more rejection), the warmth scale is reverted. Participants indicated how well each statement described their experience of remembered early care using a four-point Likert scale (from 4 = *almost always true* to 1 = *almost never true).* In this study, the two total scores had a good reliability (PARQmother α = 0.94; PARQfather α = 0.95).

#### Experimental task

**Fig. 1:**
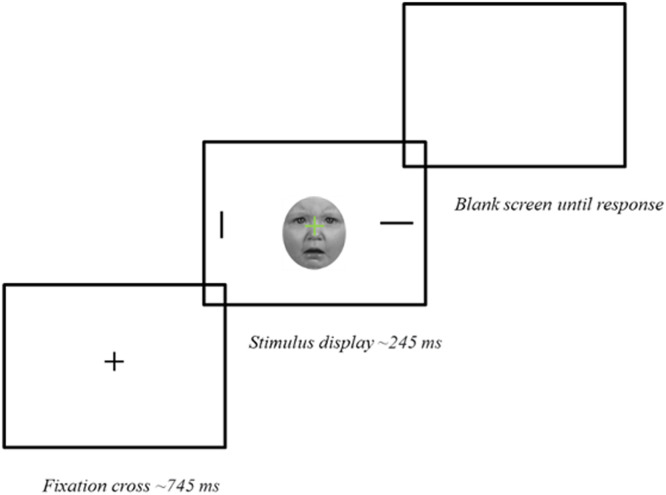
Schematic representation of a trial structure in the Go condition. Source: Gemignani et al., (2022); used with the permission of the author.

Mothers completed a modified Go/no-Go task derived from an established paradigm (Bindemann et al., 2005) to measure attentional bias to infant and adult emotional and unemotional faces (Fig. 1; Dudek & Haley, 2020; Gemignani et al., 2022; Pearson et al., 2010; 2011a; 2011b). A central black fixation point was presented for 745 milliseconds (ms). Then, the fixation point turned into green or red, signaling the Go or no-Go condition respectively. Simultaneously, two lines, one horizontal and one vertical, appeared at the periphery of the screen. Standardized images of adult and infant faces appeared behind the Go/no-Go cross during the stimulus display (245 ms). Only for Go trials, participants were asked to indicate on which side of the screen the vertical line appeared by pressing “n” (for right) or “v” (for left) on the keyboard. The screen response was aborted if no response was registered within 2000 ms. Thirty-six images (6 males; 6 females) of unfamiliar infant faces aged 4–12 months were extracted from the Tromso Infant Faces Database (TIF; Maack et al., 2017), and 36 images of unfamiliar adult faces (6 males; 6 females) from the Karolinska Directed Emotional Faces (KDEF; Lundqvist et al., 1998). For each identity, 3 facial expressions (happy, sad, neutral) were chosen. Whilst distressed infant faces displayed an infant actively crying, happy faces displayed a smiling expression, and neutral faces displayed no expression. Images were cropped in an oval shape, converted into grayscale, and presented against a uniform white background. Images were matched for size using GNU Image Manipulation Program version 2.10.34 (GIMP. 2023). Faces averaged approximately 4 × 5 cm and were equalized for luminance and saturation using MATLAB version R2019a (MathWorks, Natick, MA, USA). Mothers completed a practice block of 12 trials with no images, then a block of 12 trials displaying faces in the background. Experimental trials consisted of 6 blocks of 36 trials (24 Go and 10 no-Go). The order of trials was randomized within blocks, but Go trials occurred twice as frequently as no-Go trials. The experimental conditions were fixed for each block (see Palermo & Rhodes, 2003), but block order was randomized across participants. The target line location was balanced within each block (50 % on the right; 50 % on the left). Attentional bias was measured by calculating RTs in ms to identify the location of the target vertical line during Go trials from the onset of the stimulus display.

#### Procedure

To overcome geographical barriers in recruiting the sample, all the experimental procedures were conducted online. Self-reports were administered through Qualtrics (Qualtrics, Provo, UT). The experimental task was run on JATOS (Lange et al., 2015). The task was conducted by an experimenter during a Zoom meeting. Mothers within each couple completed the task during a single session, but they did in separate sessions whenever the first option was not applicable. Mothers were asked to keep their left index finger on the “v” and their right index finger on the “n” of the keyboard during the task. After explaining the instructions and solving doubts, experimenters shut down their microphone and camera but monitored participants’ engagement with the task.

#### Data analysis

Paired *t*-tests were run to check for potential differences within the sample. Correlation analyses were implemented. Missing data in self-reports was not replaced. Analyses were conducted for evaluating the number of correct answers for different blocks. The overall accuracy for Go trials was 96.6 %, which confirmed the ability of participants to complete the task as instructed. The percentage of false alarms (i.e., incorrect No-Go trials) were 3.9 %. Response accuracy was analyzed using a generalized mixed-effects model. RTs were analyzed via linear mixed-effects models (Bates et al., 2015); only correct trials were considered. The models were performed using the lme4 (Version 1.1-28) library (Bates et al., 2015) in Rstudio (Version 4.1.1; RStudio Team, 2021). Those responses which were too fast (below 100 ms) or longer than 1400 ms from the stimulus onset (0.2 %) were considered outliers and removed. To approximate a normal distribution, RTs were transformed into logarithms, and the distribution was checked visually on the trial-, participant- and item- levels. As the distributions were approximately normal, we did not exclude any further items, participants, or trials. Face age and emotional valence were contrast-coded, such that the intercepts reflected the grand mean for all conditions. The scores of metarnal involvement, PARQmother and PARQfather were centered by subtracting the overall mean across participants. In order to disentangle the role of maternal involvement in childcare from the concomitant contribution of other current caregiving experiences, we added the child age and parity as potential covariates in Model 2 (Table 2). In fact, several factors might be linked to present parental experience, such as the time spent with childcare (i.e., quality and quantity of activities accomplished), the duration of motherhood (i.e., child age), and the number of children that mothers have (i.e., parity). We did not include any other covariates in the models. Table 2 summarizes the aims, independent variables, random effect structures, and significant results of the main models.

**Table 2:** Overview of the aims, independent variables, random effect structures, and significant results of the main models.

Aims	Independent variables	Random effect structure	Results
**Model 1**: confirm that infant faces engaged more attention (slower RTs) compared to adult faces	Face age, emotional valence, and their interaction	Participant, stimuli	**Main effect of face age**: infant faces, compared to adult faces, retained more attention
**Model 2**: investigate whether the attentional bias to infant faces was associated with maternal involvement in childcare	Face age, maternal involvement, and their interaction. Child age and parity were added as covariates	Participant, stimuli	**Main effect of face age**: infant faces engaged more attention compared to adult faces **Two-way interaction effect between face age and maternal involvement in childcare**: more involved mothers were more biased, in terms of attention, toward infant versus adult faces
**Model 3**: investigate whether differences in past care experiences with one's own mother (PARQmother) were associated with the attentional bias to infant faces	Face age, PARQmother, and their interaction	Participant, stimuli	**Main effect of face age**: infant faces engaged more attention compared to adult faces
**Model 4**: investigate whether differences in past care experiences with one's own father (PARQfather) were associated with the attentional bias to infant faces	Face age, PARQfather, and their interaction	Participant, stimuli	**Main effect of face** age: infant faces engaged more attention compared to adult faces **Two-way interaction effect between face** age **and PARQfather**: those mothers who felt more rejected by their own father during childhood were more biased, in terms of attention, to infant versus adult faces

## Results

### Preliminary results

Table 1 represents the characteristics of the study participants in terms of means or percentages and standard deviations. Mothers reported to be statistically significantly more rejected by their own fathers than by their own mothers during childhood (*t* = 3.49, *df* = 71, *p* <.01). Pearson correlation analyses did not show any significant associations between maternal involvement and PARQmother or PARQfather. PARQmother and PARQfather showed a low positive correlation (*r* = 0.26, *p* = .03).

### Main analysis

**Fig. 2:**
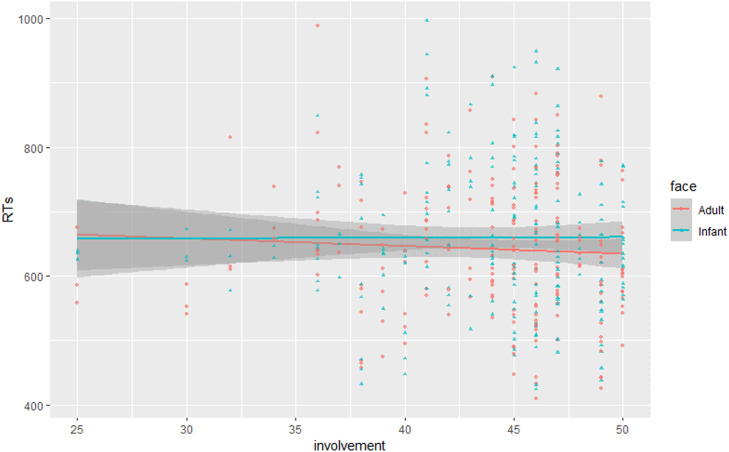
Interaction effect between face age and maternal involvement with childcare.

**Fig. 3:**
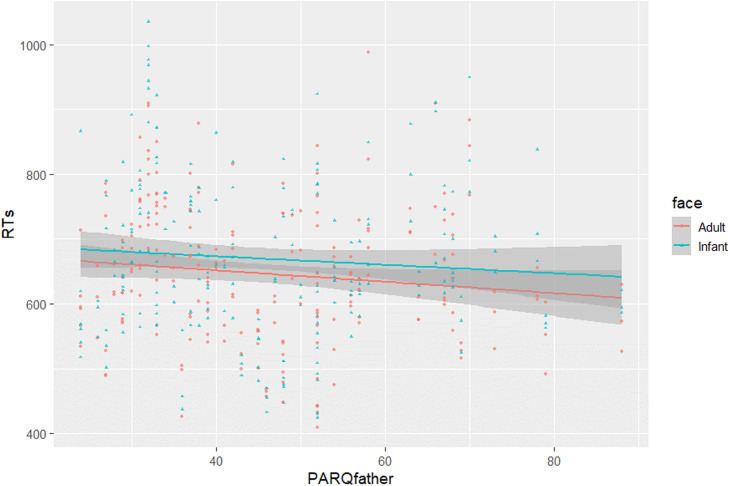
Interaction effect between face age and PARQfather.

We fitted a generalized mixed-effect model with face age and emotional valence predicting trial-level accuracy. Due to a high level of accuracy, the analysis did not yield any significant result; thus, all subsequent models used RTs as dependent variable. To investigate the first aim of this study, we implemented a linear mixed-effect model (Model 1; Table 2) in which face age (adult, infant) and emotional valence (happy, neutral, sad) were used as fixed terms, and their interaction was considered. The model included random intercepts for participants and experimental stimuli. Model 1 showed a main effect of face age (*β* = −0.015, *SE* = 0.002, *t* = −9.070, *p* < .001), as infants slowed RTs to a greater extent compared to adult faces. This effect remained statistically significant after increasing the complexity of the random effect structure by varying all the slopes (*β* = −0.015, *SE* = 0.004, *t* = −3.540, *p* < .01) and only the slope of the main effects (*β* = −0.015, *SE* = 0.004, *t* = −3.566, *p* < .01). Model 1 also evidenced a main effect of emotional valence (*β* = −0.006, *SE* = 0.002, *t* = −3.068, *p* = .003), as mothers allocated greater attention to sad faces compared to happy and neutral faces. However, this effect was not statistically significant in all the subsequent models. The interaction between face age and emotional valence was not significant. In Model 2 (Table 2), maternal involvement was added as a fixed effect in addition to face age. The interaction between the two terms was also considered. To reduce the complexity of the model, we collapsed across the expressions. Model 2 confirmed the main effect of face age, with greater attention retained by infant versus adult faces (*β* = −0.015, *SE* = 0.002, *t* = −8.011, *p* <.001). It highlighted a two-way interaction effect between face age and maternal involvement (*β* = −0.001, *SE* = 0.0003, *t* = −3.331, *p* <.001). Interestingly, more involved mothers were more biased, in terms of attention, toward infant versus adult faces (Fig. 2). Child age and parity were included as covariates in Model 2, but they did not test statistically significant. In Model 3 and Model 4 (Table 2) PARQmother and PARQfather were added as fixed effects, respectively, in addition to face age. The interaction between the terms were considered. Model 3 confirmed the main effect of face age, with greater attention retained by infant versus adult faces (*β* = −0.015, *SE* = 0.002, *t* = −8.536, *p* <.001). The interaction effect between face age and PARQmother was not significant. Model 4 confirmed the main effect of face age (*β* = −0.015, *SE* = 0.002, *t* = −8.875, *p* < .001) and highlighted a two-way interaction between face age and PARQfather (*β* = −0.0003, *SE* = 0.0001, *t* = −2.788, *p* = .005). That is, those mothers who felt more rejected by their own father were more biased, in terms of attention, by infant versus adult faces (Fig. 3). Numerical values related to the main models are reported in the Supplementary Materials.

## Discussion

This study has sought to investigate whether an enhanced attention to infant over adult faces is associated with the past care experiences with one's own caregivers during childhood and current commitment in childcare in same-sex mothers. By extending previous findings based on heteronormative samples, our study is the first one to support that the attentional prioritization of infant faces is related to the engagement with childcare in a population of mothers that is less susceptible to the traditional division of childcare ascribable to socio-cultural factors. Given that the attentional bias to infant faces has been associated with the quality of mother-infant bonding (Dudek & Haley, 2020; Pearson et al., 2011a), our results might clarify underlying mechanisms contributing to maternal sensitivity in diverse family contexts, ultimately supporting child health outcomes.

Consistent with previous research (Pearson et al., 2010; 2011a; 2011b; Thomson-Booth et al., 2014a; 2014b), we demonstrated that infant faces elicited more attention when compared to adult faces. It has been assumed that there is an intrinsic quality of infant faces (*Kinderschema*; Lorenz, 1943) which facilitates parents’ allocation of attention toward them (Oliveira et al., 2017). In an ecological perspective, an increased recruitment of attentional resources to infant cues may benefit human caregiving especially in parents, helping to respond to the infants’ needs (Ainsworth 1970; 1978). Considering that previous research has been mostly focused on heteronormative samples of mothers, our findings extended previous knowledge by including same-sex mothers.

An interaction effect between face age and emotional valence, as it was detected in previous studies (i.e., for distressed infant faces; Pearson et al., 2010; 2011a; 2011b), was not found here. However, given that this effect was not consistently evidenced in other studies (e.g., Dudek & Haley., 2020; Gemignani et al., 2022; Oliveira et al., 2017), our results might align with the strong motivational salience attributed to all infant stimuli regardless of their facial expressions. As previously suggested (Dudek & Haley., 2020), whilst the attentional bias to infant faces might be modulated by a general preferential processing of infant faces, a neural preference reflecting face–sensitive encoding might be more specific for infant distress cues. In addition, a main effect of the emotional valence emerged in the first model, such that sad faces were associated with the longest RTs compared to other conditions; however, this effect was not confirmed in subsequent models.

We demonstrated that the attentional bias to infant versus adult faces varied in relation to maternal involvement with childcare; more involved mothers were more biased, in terms of attention, to infant versus adult faces. From a visual inspection of data, it should be noted that whilst infant faces engaged mothers’ attention no matter their level of involvement, maternal attention to adult faces dropped dramatically by increasing their levels of commitment in childcare. This resulted in a greater bias to infant versus adult faces for those mothers who were more involved in childcare. The fact that we included a sample of same-sex mothers gives strength to our findings, as we ruled out socio-culturally driven differences in the division of childcare which might be found in different-sex couples. Even though previous evidence suggest that maternal attentional bias to infant cues is established prenatally and thus partially independent of caregiving experience (Dudek & Haley, 2020; Pearson et al., 2010), here we found that maternal involvement may come into play in modulating attentional bias to infant cues postnatally, with greater involvement in childcare reflecting a prioritization of attention to infant over other social stimuli. Overall, both human and animal studies suggest that parental neurobiology is experience-sensitive (e.g., Abraham et al., 2014; Stolzenberg & Champagne, 2016). As demonstrated in studies including non-biological mothers (Bick et al., 2013; Grasso et al., 2009), a preferential elaboration of infant faces is not limitedly related to biological processes, but partially related to nurturing experiences. For instance, Bick and colleagues (2013) demonstrated that foster mothers’ oxytocin production became associated with electrophysiological activity in response to mothers’ own child over the course of mother-infant bond. It should be noted that seminal studies on this topic (Abraham et al., 2014; Bick et al., 2013; Grasso et al., 2009) adopted images or video-clips of parents’ own infants as experimental stimuli. Although research on parity has demonstrated that nurturing experiences might be linked to maternal responses also to unfamiliar infant cues (Maupin et al., 2019; Rutherford et al., 2019), it might be that, whilst unfamiliar infant faces engage the attention of mothers no matter their level of involvement, a higher level of commitment in childcare would be related to an increase of maternal RTs to own infant faces. Overall, our results empathize the importance of same-sex mother's involvement in childcare, which might be related to cognitive mechanisms underlying human caregiving (i.e., attentional bias to infant faces) and ultimately linked to a positive mother–child relationship (Golombok et al., 2023).

As the measure from Abraham and colleagues’ work (2014) has been considered an optimum point of reference for assessing caregiving involvement (Giannotti et al., 2022b), we decided to extract a pool of the related questions. However, we considered only the items assessing nurturing maternal behaviors on a daily basis, which could be accomplished by both recognized and unrecognized parents in the Italian context. Those nurturing behaviors are frequently covered in parental involvement measures (for a review, see Giannotti et al., 2022b). For instance, Laflamme et al. (2002) investigated parents’ responsibility in caregiving activities related to feeding, bathing, and dressing the child, reading to the child, and going for a walk with the child. Gettler and colleagues (2011) examined the quantity of time fathers engaged in caregiving behaviors, including feeding and bathing the child, playing with the child, and reading to the child. Nonetheless, our selected questions might have provided a circumscribed assessment of parental committed behaviors, not capturing the overall dynamics of mother-child relationships across time. In addition, the contribution of other components of parental involvement, such as the emotional engagement with one's own child (e.g., soothing the child when they are upset), has been neglected in our study.

From the attachment theory perspective (Bowlby, 1969/1982), the intrapsychic organization of attachment influences the ways in which people process emotional information. With respect to the underlying processes, this may be noticeable on the differential allocation of attention toward different types of affective social stimuli (Edelstein & Gillath, 2008). In the present study, we found a significant interaction effect between early care experiences with one's own father and the attentional bias to infant faces; unexpectedly, those mothers who felt more rejected by their own father were more biased, in terms of attention, to infant compared to adult faces. We must clarify that whilst the level of attention to faces generally decreased by increasing the level of paternal rejection, attention to adult faces dropped significantly. In contrast with previous evidence (Gemignani et al., 2022), this resulted in a greater bias to infant versus adult faces in those mothers who were more rejected by their own father. Of note, the descriptive mean of paternal rejection in our sample (*M* = 46) was greater than the ones previously reported in other studies (e.g., *M* = 38.695; Gemignani et al., 2022; *M(wave1)* = 35.81, *M(wave2)* = 34.00 and *M(wave3)* = 33.52; Putnick et al., 2015). Given that a milder level of paternal rejection was reported in previous studies, a failure in finding such a result before might have been due to a lower variance in the model estimation. It should be also noted that, beyond the consideration of attentional bias as a differential measure, mothers’ RTs decreased in response to all the types of faces as the level of paternal rejection increased. Thus, in line with previous evidence showing a perceptual bias toward social information in secure versus insecure mothers (Fraedrich et al., 2010; Leyh et al., 2016), our result might importantly suggest that attention to faces generally decreased in those mothers who felt more rejected by their own father.

Contrary to our hypothesis, we did not find that attentional prioritization of infant over adult faces varied in relation to early care experiences with one's own mother. Compared to previous studies (Gemignani et al., 2022), it should be noted that our sample was smaller in size and included only women. In addition, in this study, mothers may have had different attachment styles related to adverse care experiences compared to previous research. In fact, whilst anxiously attached individuals become highly sensitive and vigilant to potential threat information and devote more cognitive resources to attachment-related material (Gillath et al., 2005), avoidantly attached individuals tend to elaborate less on the emotional cues they encode (Edelstein & Gillath, 2008). So, differences in attachment styles might explain a different deployment of attention toward social cues in our sample. In addition, considering that previous evidence was based on heteronormative samples, the experience of parental rejection itself might somewhat differ among sexual minorities, and they might be more exposed to adverse care experiences as compared to heterosexual identities (Fuller, 2017).

## Limits and future directions

Some limitations of our study should point toward future directions. Future research might clarify the role of individual mental health as a potential covariate in relation to the behavioral findings (e.g., Pearson et al., 2010). In non-heterosexual identities, the role of perceived stigma might also require greater attention (Baiocco et al., 2015). Moreover, data from mothers being in a couple may share some sort of dependencies, which could be explored in larger samples by adopting the Actor–Partner Interdependence Model (APIM; Cook & Kenny, 2005). Different routes to parenthood for same-sex mothers have not been analyzed in this study; indeed, this might have exposed a great variability in our conclusion. On this note, we collected the information regarding the type of relation that mother had with the child, such that mothers could pick one of the following options (i.e., biological parent, adoptive parent, step-parent, foster parent, non-biological parent recognized at birth, prefer not to say, other) or self-define themselves. However, since this was not one of the main aims of our study, we refrained from asking further information about the conception of the child, as this may have added vulnerability to our sample of same-sex mothers (Wells, & Lang, 2016). Overall, given the wide variability of the information available, we decided that it would not be appropriate to dichotomize the variable into distinctive categories (e.g., biological versus non-biological parents). However, future research could focus on this topic embracing the complexity of different types of mother–child relationships, paying much attention to the new reproductive options (i.e., ROPA, Reception of Oocytes from the Partner), to take those factors into account also in the statistical models. Importantly, investigating the role of biological relatedness with the child might strengthen the idea that biological processes might explain only partially maternal responsiveness to infant cues, and that maternal involvement is important for mother–infant bondings besides the experience of pregnancy and birth (Golombok et al., 2023). Moreover, since infants’ facial expressions might convey important information for understanding infants’ physical and mental needs, further research should explore more in depth the role of the emotional valence of facial expressions in maternal responses to infant faces. By adopting longitudinal designs, further research is also needed to establish the direction of the associations found in this study. In doing so, researchers should be warned that answers about the childcare activities provided by same-sex mothers may be biased by structural barriers (i.e., relating to laws and policies) which might be present in each country. For instance, in Italy, some childcare activities cannot be automatically accomplished by those parents unrecognized as legal guardians of the child. In our study, we were therefore careful to consider only those items covering parental behaviors which could be accomplished by both recognized and unrecognized mothers. With respect to the parental involvement measure, further studies with a larger number of participants should investigate the expected unifactorial structure of the measure selected in our study, in order to ascertain whether it might be reliable or not. Otherwise, a gold-standard and inclusive measure to investigate parental involvement in childcare in the Italian context might be developed in future (Giannotti et al., 2022b). Another difficulty in this field is that same-sex parent samples mainly involve individuals with a high or medium-high socio-economic status, as they need to afford the often expensive medical treatments to become parents. Further research should consider less visible parents (e.g., transgender parents) and different intersectionalities (e.g., different ethnic groups, social classes). With regard to previous experiences of care during childhood, further research should distinguish between early experiences of care with primary/secondary caregivers, rather than asking about past experiences with one's own mother/father. Such methodology could sound as an inclusive practice in research. This would also pair with the postulates of IPARTheory, according to which a significant other is defined as any person with whom a child has a relatively long-lasting emotional tie, independently of the biological relationship (Rohner et al., 2016). Eventually, we are aware that parenthood is a complex phenomenon, and multiple factors might contribute to caregiving differences above and beyond cognitive variations related to attention; therefore, it is essential to examine whether attentional bias might constitute an early determinant of maternal sensitivity in same-sex mothers in order to ultimately promote maternal sensitivity and child development.

## Conclusion

In a considerable sample of same-sex mothers, we examined the contribution of past and current experiences of care on the attentional bias toward infant faces. Taken together, mental representations of care built during childhood and direct commitment in childcare were associated with same-sex mothers’ attentional bias toward infant faces. Apart from having a value on its own, inclusive research of different family contexts is needed conceptually and methodologically, as this might allow researchers to move beyond sex-related differences in families and consider different roles and arrangements (Carone & Lingiardi, 2022). Overall, psychological research needs to embrace the complexity of nowadays plural family models, socializing the idea that there are different ways of conceiving and understanding parenting (Monaco & Nothdurfter, 2023). On this note, this work represents an opportunity to extend previous knowledge confined to different-sex parents. Importantly, understanding factors that compromise maternal responses to infant signals may aid in creating tailored interventions for vulnerable mothers in different family contexts. Quantitative synthesis consistently indicated positive outcomes for children raised by same-sex families (Fedewa et al., 2015); however, perceived social stigma has been found to negatively interfere with child adjustment (Bos & van Balen, 2008; Golombok et al., 2018). We crucially point to the need of framing research on same-sex parent families for accurate and robust empirical findings to inform social policies aimed at reducing social stigma and promoting the well being of these families.

## Declaration of Competing Interest

The authors declare that they have no known competing financial interests or personal relationships that could have appeared to influence the work reported in this paper.

## Data Availability

The data that support the findings of this study are available from the corresponding author upon reasonable request.

## Appendix Supplementary materials

Image, application 1
