# Supplementary material for: Attentional bias to infant faces might be associated with previous care experiences and involvement in childcare in same-sex mothers
Source: Int J Clin Health Psychol. 2023 Oct 23;24(1):100419. doi: 10.1016/j.ijchp.2023.100419 (PMC10598538; doi:10.1016/j.ijchp.2023.100419)
Supplement: Supplementary file 1 [file mmc1.docx]

**Items on parental nurturing behaviors**

1) How often did you change your child’s diaper?

2) How often did you prepare meals or bottles for your child?

3) How often did you feed your child or give your child a bottle?

4) How often did you play peek-a-boo with your child?

5) How often did you hold them?

6) How often did you do things like tickle your child, blow on their belly, or move their arms and legs around in a playful way?

7) How often did you put your child to sleep?

8) How often did you wash or bath your child?

9) How often did you take your child outside for a walk or to play in the yard, a park or a playground?

10) How often did you dress your child?

**(5-More than once a day/ 4-About once a day/ 3-a few times a week/ 2-Rarely/ 1-Not at all)**

**Results of the Main Models**

**Results of Model 1**

| **Fixed effect** | **β** | **SE** | **t** | **p** |
| --- | --- | --- | --- | --- |
| Face age | -0.015 | 0.002 | -9.070 | <.001 |
| Emotional valence | -0.006 | 0.002 | -3.068 | .003 |
| Face*Emotion | 0.003 | 0.002 | 1.297 | .2 |

**Note:** The main effect of emotional valence did not remain stable in the following models. RTs were log-transformed; all the other details regarding variables are reported in the manuscript; SE= Standard Error.

**Results of Model 2**

| **Fixed effect** | **β** | **SE** | **t** | **p** |
| --- | --- | --- | --- | --- |
| Face age | -0.015 | 0.002 | -8.011 | <.001 |
| Involvement | -0.001 | 0.004 | -0.268 | .8 |
| Face*Involvement | -0.001 | 0.0003 | -3.331 | <.001 |
| Child age | -0.00003 | 0.002 | -0.015 | .99 |
| Parity | -0.02 | 0.06 | -0.411 | .7 |

**Note:** RTs were log-transformed; all the other details regarding variables are reported in the manuscript; SE= Standard Error.

**Results of Model 3**

| **Fixed effect** | **β** | **SE** | **t** | **p** |
| --- | --- | --- | --- | --- |
| Face age | -0.015 | 0.002 | -8.536 | <.001 |
| PARQmother | 0.0001 | 0.001 | 0.035 | .97 |
| Face*PARQmother | -0.00001 | 0.0001 | -0.098 | .9 |

**Note:** RTs were log-transformed; all the other details regarding variables are reported in the manuscript; SE= Standard Error.

**Results of Model 4**

| **Fixed effect** | **β** | **SE** | **t** | **p** |
| --- | --- | --- | --- | --- |
| Face age | -0.016 | 0.002 | -8.875 | <.001 |
| PARQfather | -0.001 | 0.002 | -0.912 | .4 |
| Face*PARQfather | -0.0003 | 0.0001 | -2.788 | .005 |

**Note:** RTs were log-transformed; all the other details regarding variables are reported in the manuscript; SE= Standard Error.
